# A whole genome SNP genotyping by DNA microarray and candidate gene association study for kidney stone disease

**DOI:** 10.1186/1471-2350-15-50

**Published:** 2014-05-02

**Authors:** Nanyawan Rungroj, Choochai Nettuwakul, Nirinya Sudtachat, Oranud Praditsap, Nunghathai Sawasdee, Suchai Sritippayawan, Duangporn Chuawattana, Pa-thai Yenchitsomanus

**Affiliations:** 1 Division of Molecular Medicine, Department of Research and Development, Faculty of Medicine Siriraj Hospital, Mahidol University, Bangkok 10700, Thailand; 2 Division of Molecular Genetics, Department of Research and Development, Faculty of Medicine Siriraj Hospital, Mahidol University, Bangkok, Thailand; 3 Medical Biotechnology Unit, National Center for Genetic Engineering and Biotechnology (BIOTEC), National Science and Technology Development Agency (NSTDA), Bangkok, Thailand; 4 Department of Immunology and Immunology Graduate Program, Faculty of Medicine Siriraj Hospital, Mahidol University, Bangkok, Thailand; 5 Division of Nephrology, Department of Medicine, Faculty of Medicine Siriraj Hospital, Mahidol University, Bangkok, Thailand

**Keywords:** Kidney stone disease, Nephrolithiasis, Genetic association study, Single nucleotide polymorphisms, Candidate gene

## Abstract

**Background:**

Kidney stone disease (KSD) is a complex disorder with unknown etiology in majority of the patients. Genetic and environmental factors may cause the disease. In the present study, we used DNA microarray to genotype single nucleotide polymorphisms (SNP) and performed candidate gene association analysis to determine genetic variations associated with the disease.

**Methods:**

A whole genome SNP genotyping by DNA microarray was initially conducted in 101 patients and 105 control subjects. A set of 104 candidate genes reported to be involved in KSD, gathered from public databases and candidate gene association study databases, were evaluated for their variations associated with KSD.

**Results:**

Altogether 82 SNPs distributed within 22 candidate gene regions showed significant differences in SNP allele frequencies between the patient and control groups (*P* < 0.05). Of these, 4 genes including *BGLAP*, *AHSG*, *CD44*, and *HAO1,* encoding osteocalcin, fetuin-A, CD44-molecule and glycolate oxidase 1, respectively, were further assessed for their associations with the disease because they carried high proportion of SNPs with statistical differences of allele frequencies between the patient and control groups within the gene. The total of 26 SNPs showed significant differences of allele frequencies between the patient and control groups and haplotypes associated with disease risk were identified. The SNP rs759330 located 144 bp downstream of *BGLAP* where it is a predicted microRNA binding site at 3′UTR of *PAQR6* – a gene encoding progestin and adipoQ receptor family member VI, was genotyped in 216 patients and 216 control subjects and found to have significant differences in its genotype and allele frequencies (*P* = 0.0007, OR 2.02 and *P* = 0.0001, OR 2.02, respectively).

**Conclusions:**

Our results suggest that these candidate genes are associated with KSD and *PAQR6* comes into our view as the most potent candidate since associated SNP rs759330 is located in the miRNA binding site and may affect mRNA expression level.

## Background

Kidney stone disease (KSD) is a major clinical problem causing medical care expenses and public health burden worldwide. The etiology of kidney stone is heterogeneous, ranging from monogenic defect to complex interaction between genetic and environmental factors [1]. KSD is endemic in the northeastern (NE) population of Thailand. The most recent investigation from our group demonstrated that it is indeed prevalent in this population and the risk of having the disease is higher among members of affected families, compared to that of the normal control population, indicating an involvement of a genetic factor, although its actual etiology is still unidentified [2].

The technology to identify genetic polymorphisms, especially single nucleotide polymorphism (SNP), is available as a tool for studying genes associated with many diseases [3,4]. Genetic contribution to kidney stone formation has been well recognized, and a number of studies have reported genetic variations of several human genes, including *osteopontin (OPN)*[5,6], *calcitonin receptor (CTR)*[7], *vitamin D receptor (VDR)*[8], *urokinase*[9], *interleukin (IL-1β and IL-Ra)*[10,11], *E-cadherin*[12], *androgen-oestrogen receptors (AR and ER)*[13], *vascular endothelial receptor growth factor (VEGF)*[14], and *calcium-sensing receptor (CaSR)*[15], which associated with KSD with a predominantly hypercalciuria and calcium oxalate kidney stones. However, these reported genes had little effect on clarifying the contribution to KSD and a frequent genetic abnormality causing the disease has not been elucidated. Two previous genome-wide association studies (GWAS) examined the whole genomic DNA with many thousands of SNPs and reported genes that were associated with KSD. Sequence variations in *claudin 14 (CLDN14)* gene associated with KSD in the population from Iceland and the Netherlands have been reported [16]. Subsequently, the same research group described that *uromodulin (UMOD)* variant affected risk of chronic kidney disease by providing protection against KSD [17].

To investigate genetic variations associated with KSD in NE Thai population, our group has previously reported the result of case–control association study in 112 subjects each of patient and control groups. We genotyped 67 SNPs distributed within 8 candidate genes, namely *TFF1*, *S100A8*, *S100A9*, *S100A12*, *AMBP*, *SPP1*, *UMOD*, and *F2*, that encode stone inhibitor proteins, including trefoil factor 1, calgranulin (A, B, and C), bikunin, osteopontin, Tamm-Horsfall protein, and prothrombin, respectively. We found that minor allele and homozygous genotype frequencies of 8 from 10 analyzed SNPs distributed within the *F2* gene were significantly higher in the control group than those in the patient group. In addition, two *F2* haplotypes were found to be dually associated with kidney stone (one with decreased risk and the other with increased risk) in the female group, indicating that *F2* variations influence the risk of KSD in the NE Thai female patients [18]. We further examined and reported the association between a specific *F2* variation (T165M) and KSD in our recent work [19].

The genes that have been reported for their association with KSD in other populations may serve as candidate genes for initial screening in the population of interest that is affected by this disease. In this study, we initially carried out a whole genome SNP genotyping by DNA microarray. The genetic variations associated with KSD in the NE Thai population were then determined by candidate gene association study using data of the reported candidate genes involved in KSD. The result showed that at least four candidate genes were associated with KSD in the NE Thai population studied.

## Methods

### Subjects

The study was approved by Siriraj Institutional Review Board and the Ethics Committees of the Ministry of Public Health, Thailand. A written informed consent was obtained from individual subject before conducting this study. A group of 216 patients with KSD (135 females and 81 males, aged 22–80 years), recruited from Khon Kaen Regional Hospital in the northeastern part of Thailand, were studied. Of these, 64% have family history. The 216 healthy control subjects (126 females and 90 males), who were age-matched, unrelated, and had no history of KSD, were recruited from the same geographical region. More details of the patients and the healthy control subjects as well as the diagnostic procedures have been described in our previous report [19]. Genomic DNA samples of the patients and control subjects were extracted from peripheral blood samples using the standard phenol-chloroform method.

### Genotyping

The genomic DNA samples from 101 patients and 105 normal control subjects were genotyped by using the Affymetrix Genome-Wide Human SNP Array 6.0 and following the manufacturer’s instructions (http://www.affymetrix.com/support/technical/byproduct.affx?product=genomewidesnp_6). The genotyping was conducted by Vanderbilt Functional Genomics Shared Resource (Vanderbilt University, USA). Briefly, genomic DNA (500 ng) was digested with *Nsp* I and *Sty* I restriction enzymes and ligated to adaptors. A single generic primer that recognizes the adaptor sequence was used to amplify adaptor-ligated DNA fragments. PCR amplification products for each restriction enzyme digest were combined and purified using polystyrene beads. The amplified products were then fragmented, labeled, and hybridized to a SNP Array 6.0. Genotyping Console™ Software was used for genotype calling.

### Candidate gene search

Candidate genes for KSD were searched and chosen for the study and statistical analysis from data sources presented during 1990–2011. Traditional or manual literature search was carried out using PubMed database (http://www.ncbi.nlm.nih.gov/pubmed/). Two additional resources, SNPs3D (http://snps3d.org/) [20] and HuGE Navigator (http://www.hugenavigator.org/HuGENavigator/home.do) [21], were also used to assist in screening candidate genes from published human genetic association studies using the keywords related to KSD. Candidate genes were selected according to the following criteria: encoding protein involving in KSD (i.e. ion exchange or ion channel protein), stone promoter or inhibitor proteins, proteins involving pathways of calcium, kidney acid–base homeostasis proteins, and metabolic enzymes.

### Determination of SNPs in candidate gene regions

SNPs in candidate gene regions (including 1 kb upstream and 1 kb downstream of transcript position) from the genotyping result of 101 case and 105 control subjects were determined for their association with KSD. SNPs with call rate of genotyping less than 90% were excluded. Candidate genes that showed significant differences of SNP allele frequencies between the patient and control groups were further assessed for their involvement in the disease by analyses of their individual SNPs and haplotypes.

### Sequencing

Specific primers for PCR amplification and sequencing in the regions of promoter, all exons, and exon-intron boundaries of *BGLAP* and *AHSG* genes were designed by Primer3 program (http://frodo.wi.mit.edu/primer3/) (the sequences of PCR primers are available on request). PCR amplification and sequencing were performed in 6 patients and 6 control subjects. The PCR was performed using the Immolase™ DNA polymerase (Bioline, USA) with 0.5 pmol each of forward and reverse primers, 1x Immolase buffer, 1.5-3 mM MgCl_2_ and 100 ng of genomic DNA. The cycling conditions were set as follows: one cycle at 94°C for 10 min, 30 cycles of denaturation at 94°C for 30 sec, annealing at 64-67°C for 1 min, and extension at 72°C for 45 sec.

The PCR products were purified for direct sequencing by using ExoSAP-IT® (Affymetrix, USA). All sequencing PCR reactions were conducted under BigDye™ Terminator Cycling conditions using 3730XL DNA analyzer (Applied Biosystems™, USA) by Macrogen Inc (South Korea). The sequences were evaluated and compared with the reference nucleotide sequences of *BGLAP* (NC_000001.10) and *AHSG* (NC_000003.11) by multiple sequence alignment using ClustalW2 program (http://www.ebi.ac.uk/Tools/clustalw2/index.html).

### SNP genotyping by a high-resolution melting method

Three SNPs located in *BGLAP* gene region and the adjacent *PAQR6* gene (a novel SNP in exon 3, rs759330, and rs7513351) and two SNPs (rs4917 and rs4918) of *AHSG* were genotyped by high-resolution melting (HRM) method. PCR and HRM analysis for these SNPs were prepared in 384-well plate and performed on a LightCycler 480 II machine (Roche Diagnotics, Germany). All PCR reactions were optimized by performing reactions in 20 μL volumes containing 125 ng genomic DNA in 1x Immolase buffer, 0.5 μM of each primer, 0.2 mM dNTP, 1.5 mM MgCl_2_, 0.25 unit of DNA polymerase (Immolase, Bioline, USA), and 1x Resolight dye (Roche Diagnostics, Germany). The PCR condition included an initial denaturation at 95°C for 10 min, followed by 35 cycles of 95°C for 20 sec, 60-68°C for 20 sec, and 72°C for 20 sec. Before the HRM step, the product was heated to 95°C for 30 sec and then cooled to 40°C. Melting curves were obtained by increasing the temperature from 65-97°C with 25 acquisitions of continuous florescence detection. The melting curves were normalized, temperature-shifted and converted to difference plots by Gene Scan software. The sequences of PCR primers are available on request.

In some cases in which the melting curves and difference plots of amplicons from the samples with homozygous wild-type and variants were unable to be differentiated, they were further analyzed by spiking the DNA sample carrying homozygous wild-type into the tested samples to allow the detection of heteroduplex DNA molecules generated from the homozygous variant samples [22,23].

### Statistical analysis

According to the SNP genotyping data of microarray, significant difference SNPs (*P* < 0.05) between 101 patients and 105 normal control subjects were examined by GenABEL package with no adjustment for binary traits; the test is equivalent to the Armitage test [24]. Statistical tests for Hardy-Weinberg equilibrium (HWE) and for association between SNP frequencies and disease phenotype were performed by 3 web-based programs including DeFinetti (http://ihg.gsf.de/cgi-bin/hw/hwa1.pl, SNPStats (http://bioinfo.iconcologia.net/snpstats/start.htm), and Haploview (http://www.broad.mit.edu/mpg/haploview/). *P*-values were calculated using a chi-square test or Fisher’s exact test. *P* < 0.05 was considered statistically significant in the calculation to compare their differences. The Haploview software package was used to estimate the pair-wise linkage disequilibrium (LD) and haplotype block structure using the confidence intervals algorithm. Bonferroni’s correction for multiple testing was applied by multiplying *P*-value by the number of observed haplotypes.

## Results

### Candidate genes for kidney stone disease

To identify and collect candidate genes involved in KSD, all published articles or abstracts in the genetic studies of KSD and its genetic association studies were searched both from the PubMed database and by using SNPs3D and HuGE Navigator. Altogether 104 candidate genes (97 genes on autosome and 7 genes on chromosome X) were gathered: 48 genes from manual literature search from PubMed, 64 genes from SNPs3D, and 49 genes from Phenopedia of HuGE Navigator (Additional file 1: Table S1).

### Potential candidate genes with significant SNPs

A total of 1,559 SNPs located within 104 candidate gene regions were taken from the genotyping results analyzed by using Affymetrix Genome-Wide Human SNP Array 6.0 of the patient and normal control groups and were primarily evaluated for their variations associated with KSD. Several criteria were considered for further selection, i.e., number of analyzed SNP on microarray, number of significant SNP, ratio of significant SNP and SNP on microarray of each gene, gene expression data, and protein function information. The result demonstrated that 82 SNPs distributed within 22 candidate gene regions, 20 genes on autosome and 2 genes on chromosome X, showed significant differences of SNP allele frequencies between the patient and control groups (uncorrected *P* < 0.05) (Table 1). After Bonferroni’s correction for 1,559 comparisons, the new statistical significance cut off is *P* = 0.000032 and none of SNP can pass this requirement. Of these, 5 candidate genes, including *BGLAP*, *SLC2A9*, *EGFR*, *CD44*, and *HAO1*, had at least one SNP that showed highly significant differences of allele frequencies between the patient and control groups at *P* < 0.005. By taking the number of SNP with significant *P*-value within the gene into consideration, 4 candidate genes including *BGLAP*, *AHSG*, *CD44*, and *HAO1* which encode osteocalcin, fetuin-A, CD44 molecule, and glycolate oxidase 1, respectively, were selected to be further assessed for their involvement in KSD by analyses of individual SNPs and haplotypes because they carried high proportion of SNPs with statistical differences of allele frequencies between the patient and control groups within the gene (ClinVar database accession number: SCV00147950-66). The detail of each candidate gene is described below.

**Table 1:** Candidate genes and SNPs associated with KSD

**Gene**	**Number of SNP in gene region**			**SNP with the least *P*-value**	***P*-value**^**c**^
	**SNP on microarray**	**SNP with *P*** **< 0.05**	**SNP with *P*** **< 0.005**		
*S100A12*	1	1	0	rs4772	0.04258
*BGLAP*	1	1	1	rs759330	0.00070
*HSPG2*	30	3	0	rs16826053	0.01248
*IL1R1*	19	1	0	rs871657	0.04641
*AHSG*	3	2	0	rs2070635	0.02556
*CLDN16*	8	1	0	rs17504970	0.00651
*SLC2A9*	122	21	1	rs939134	0.00428
*SLC26A2*	6	1	0	rs245081	0.03632
*EDN1*	10	2	0	rs2070699	0.02020
*EGFR*	92	2	2	rs7801956	0.00958
*CALCR*	54	1	0	rs13232226	0.03644
*SLC26A5*	33	1	0	rs17142677	0.04889
*CAV1*	19	2	0	rs6867	0.03657
*CD44*	52	12	1	rs353647	0.00298
*F2*	6	1	0	rs2070852	0.01117
*VDR*	39	4	0	rs2254210	0.01815
*RASGRP1*	21	7	0	rs7177101	0.00701
*ANXA2*	23	4	0	rs7183894	0.03945
*CDH1*	33	1	0	rs12444784	0.04469
*HAO1*	35	11	4	rs6118004	0.00179
*AR*	21	1	0	rs5031002^a^	0.02756
*HPRT1*	3	2	0	rs6634993^b^	0.04254

^a^SNP was significantly different between patients and control subjects only in male group.

^b^SNP was significantly different between patients and control subjects only in female group.

^c^Uncorrected *P*-value.

#### BGLAP

Only one SNP, rs759330, located 144 bp downstream of *BGLAP* gene was identified from the result of DNA microarray. This SNP was in HWE and showed significant differences of allele frequency between the patient and control groups with *P* = 0.0009. The analysis also revealed that genotype frequency significantly differed (*P* = 0.0027) with the model of dominant inheritance that gave the least value of Akaike’s Information Criterion (AIC). The patient group had significantly higher proportions of the combination of heterozygous genotypes of major/minor alleles (T/C) and homozygous genotypes of minor alleles (C/C) than that of the control group, indicating that C allele was susceptible to KSD with odds ratio (OR) 2.44 (Table 2). However, this association was not statistically significant after correction for 1,559 comparisons. To confirm this association, we conducted the genotyping of SNP rs759330 in 101 patients and 105 controls and the additional DNA samples of 115 patients and 111 controls to make up the total numbers of 216 each of patient and control groups by the HRM method. The differences of allele and genotype frequencies between the patient and control groups were statistically significant at *P* = 0.0001 and 0.0007 (corrected *P* = 0.0005 and 0.0035), respectively (Table 2).

**Table 2:** **Genotype and allele frequencies of SNP rs759330 in *BGLAP *** **gene**

**Genotype**	**Genotype frequency (%)**		**OR (95% CI)**	***P*-value (Corrected)**	**Allele**	**Allele frequency (%)**		**OR (95% CI)**	***P*-value (Corrected)**
	**Control**	**Patient**				**Control**	**Patient**		
SNP array	(*n = 105*)	(*n = 101*)				(*n = 105*)	(*n = 101*)		
T/T	75.2	55.5	1.00	**0.0027** (4.2093)	T	87.1	74.3	1.00	**0.0009** (1.4031)
T/C, C/C	24.8	44.5	2.44 (1.35-4.41)		C	12.9	25.7	2.35 (1.41-3.92)	
HRM	(*n = 216*)	(*n = 216*)				(*n = 216*)	(*n = 216*)		
T/T	75.0	59.7	1.00	**0.0007 (0.0035)**	T	87.0	76.9	1.00	**0.0001 (0.0005)**
T/C, C/C	25.0	40.3	2.02 (1.34-3.05)		C	13.0	23.1	2.02 (1.41-2.90)	

OR, odds ratio; CI, confidence interval.

Corrected *P*-values are adjusted for multiple comparisons (1,559 tests for SNP array and 5 tests for HRM method). Statistical significance cut off values are *P* = 0.00032 and *P* = 0.01 for the analyses by SNP array and HRM, respectively.

Significant *P*-values are indicated in bold.

To examine whether there were other SNPs linked to SNP rs759330, the whole *BGLAP* gene including its promoter region, all 4 exons and introns were amplified (as a single fragment) and sequenced in 6 patients and 6 normal control subjects. An exoinc non-synonymous SNP rs182775321 was detected. However, the results of genotyping of this SNP carried out by the HRM method in 112 of each of patient and control groups showed no significant differences of genotype and allele frequencies between the patient and control groups (*P* = 0.780 and 0.778). In addition, the results of genotyping of rs7513351 – a SNP positioned 664 bp from rs759330 which is located on *PAQR6* gene nearby the *BGLAP* – carried out by the HRM method in 112 of each of patient and control groups showed no significant differences of genotype and allele frequencies between the patient and control groups (*P* = 0.960 and 0. 0.831) (data not shown).

The Haploview software package was used to estimate the pair-wise linkage disequilibrium (LD) and haplotype block structure from the genotyping data of 3 SNPs (rs182775321, rs759330, and rs7513351) from 101 patients and 105 control subjects. The result showed that these 3 SNPs were not in LD block.

#### AHSG

Three SNPs (rs2248690, rs2070634, and rs2070635) located in *AHSG* gene region were analyzed. All analyzed SNPs were in HWE. SNP allele frequencies showed significant differences between the patient and control groups for 2 SNPs located in intron 4, rs2070634 and rs2070635 (*P =* 0.0329 and 0.0256). Analysis of individual SNPs in patient and control groups using the web-based SNPStats program revealed that genotype frequencies of rs2070634 and rs2070635 significantly differed (Table 3). The model of inheritance considered by the least value of AIC was found to be recessive for both SNPs with significant differences. The patient group had significantly higher proportions of homozygous genotypes of minor alleles than that of the control group for rs2070634 and rs2070635 (*P =* 0.023 and 0.017), indicating that homozygous genotypes of minor alleles were susceptible to kidney stone with ORs 2.49 and 2.52, respectively.

**Table 3:** **Genotype and allele frequencies of SNPs in *AHSG *** **gene observed in 105 control subjects and 101 patients**

**SNP ID**	**Genotype**	**Genotype frequency (%)**		**OR (95% CI)**	*P*-value*	**Allele**	**Allele frequency (%)**		**OR (95% CI)**	*P*-value*
		**Control**	**Patient**				**Control**	**Patient**		
rs2070634	G/G, G/T	90.5	79.2	1.00	**0.023**	G	65.7	55.4	1.00	**0.0329**
	T/T	9.5	20.8	2.49 (1.11-5.60)		T	34.3	44.6	1.54 (1.03-2.29)	
rs2070635	C/C, C/T	89.5	77.2	1.00	**0.017**	C	65.2	54.5	1.00	**0.0256**
	T/T	10.5	22.8	2.52 (1.60-5.49)		T	34.8	45.5	1.57 (1.06-2.33)	

OR, odds ratio; CI, confidence interval.

*Uncorrected *P*-value.

Significant *P*-values are indicated in bold.

One LD block spanning 6 kb containing all 3 SNPs was defined from the genotyping data of 101 patients and 105 control subjects with 3 observed haplotypes (Additional file 2: Figure S1, Additional file 3: Table S2). The most common haplotype, AGC (frequency = 0.600), was significantly more frequent in the control group (frequency = 0.652) than in the patient group (frequency = 0.545) with a *P*-value of 0.0256 (OR 0.64, 95% CI 0.43 - 0.951), indicating its association with decreased disease risk. In contrast, ATT haplotype was more represented in the patient group (frequency = 0.285) than in the control group (frequency = 0.186) with a *P*-value of 0.0170 (OR 1.743, 95% CI 1.098 -2.769), indicating its association with increased disease risk. However, after Bonferroni’s correction for 3 comparisons, the differences were not statistically significant (*P =* 0.0768 and 0.0510).

All of the 7 exons, including exon-intron boundary, and promoter region of *AHSG* gene were amplified and sequenced in 6 patients and 6 control subjects. A total of 3 exonic SNPs were detected, a synonymous SNP (rs4831) in exon 1 and two non-synonymous SNPs (rs4917 and rs4918) in exons 6 and 7, respectively. Genotyping of 2 non- synonymous SNPs, rs4917 and rs4918, was carried out by HRM method in 112 each of patient and control groups. However, no significant differences of allele and genotype frequencies between the patient and control groups of both SNPs were found (*P =* 0.203 and 0.242) (data not shown).

#### CD44

A total of 52 SNPs located in *CD44* gene region were analyzed. Three monomorphic SNPs (rs1467558, rs10488811, and rs16927100) were observed and were excluded from further statistical analysis. Allele frequencies of 12 SNPs showed significant differences (*P* < 0.05) between the patient and control groups (Table 4). Analysis of individual SNPs in the patient and control groups by using the SNPStats program revealed 11 SNPs with significant differences of genotype frequencies. In the dominant model of inheritance for these SNPs, the control group had significantly higher proportions of the combination of heterozygous genotypes of major/minor alleles and homozygous genotypes of minor alleles than that of the patient group, indicating that minor alleles were protective to KSD with ORs between 0.29 and 0.53 (Table 4).

**Table 4:** **Genotype and allele frequencies of SNPs in *CD44 *** **gene observed in 105 control subjects and 101 patients**

**SNP ID**	**Genotype**	**Genotype frequency (%)**		**OR (95% CI)**	*P*-value*	**Allele**	**Allele frequency (%)**		**OR (95% CI)**	*P*-value*
		**Control**	**Patient**				**Control**	**Patient**		
rs353623	C/C	69.2	83.2	1.00	**0.018**	C	81.2	90.6	1.00	**0.0066**
	C/T, T/T	30.8	16.8	0.46 (0.23-0.89)		T	18.8	9.4	0.45 (0.25-0.81)	
rs353618	A/A	69.5	83.2	1.00	**0.021**	A	81.4	90.6	1.00	**0.0075**
	A/G, G/G	30.5	16.8	0.46 (0.24-0.90)		G	18.6	9.4	0.45 (0.25-0.82)	
rs353612	C/C	86.7	95	1.00	**0.034**	C	92.9	97.5	1.00	**0.0275**
	C/T, T/T	13.3	5	0.34 (0.12-0.98)		T	7.1	2.5	0.33 (0.12-0.92)	
rs353637	T/T	87.1	96	1.00	**0.022**	T	93.6	98	1.00	**0.0286**
	T/A	12.9	4	0.29 (0.09-0.91)		A	6.4	2	0.30 (0.10-0.94)	
rs353630	G/G	86.7	95	1.00	**0.034**	G	93.3	97.5	1.00	**0.0426**
	G/A	13.3	5	0.34 (0.12-0.98)		A	6.7	2.5	0.35 (0.13-1.00)	
rs353647	G/G	50.5	70.3	1.00	**0.0035**	G	71.4	83.7	1.00	**0.0030**
	G/C, C/C	49.5	29.7	0.43 (0.24-0.76)		C	28.6	16.3	0.49 (0.30-0.79)	
rs3794110	C/C	74	87	1.00	**0.019**	C	87	93	1.00	**0.0446**
	C/T, T/T	26	13	0.43 (0.21-0.88)		T	13	7	0.50 (0.26-0.99)	
rs3794109	A/A	74.3	87.1	1.00	**0.019**	A	87.1	93.1	1.00	**0.0446**
	A/G, G/G	25.7	12.9	0.43 (0.21-0.88)		G	12.9	6.9	0.50 (0.26-0.99)	
rs112762	C/C	42.9	58.4	1.00	**0.025**	C	65.2	76.7	1.00	**0.0103**
	C/T, T/T	57.1	41.6	0.53 (0.31-0.93)		T	34.8	23.3	0.57 (0.37-0.88)	
rs10488809	T/T	75.2	87.1	1.00	**0.028**	T	87.6	93.1	1.00	0.0618
	T/A, A/A	24.8	12.9	0.45 (0.22-0.93)		A	12.4	6.9	0.53 (0.27-1.04)	
rs3794105	G/G	86.7	95	1.00	**0.034**	G	93.3	97.5	1.00	**0.0426**
	G/A	13.3	5	0.34 (0.12-0.98)		A	6.7	2.5	0.35 (0.13-1.00)	
rs7110737	T/T	36.2	43.6	1.00	0.28	T	58.1	67.8	1.00	**0.0411**
	T/A, A/A	63.8	56.4	0.73 (0.42-1.29)		A	41.9	32.2	0.66 (0.44-0.98)	
rs7116432	C/C	43	55.2	1.00	0.087	C	65	74.5	1.00	**0.0413**
	C/T, T/T	57	44.8	0.61 (0.35-1.08)		T	35	25.5	0.64 (0.41-0.98)	

OR, odds ratio; CI, confidence interval.

*Uncorrected *P*-value.

Significant *P*-values are indicated in bold.

Seven LD blocks containing 8, 3, 2, 2, 6, 3 and 4 SNPs were defined from the genotyping data of 101 patients and 105 control subjects with 4, 3, 3, 2, 5, 4 and 3 observed haplotypes for blocks 1–7 (Additional file 4: Figure S2 and Additional file 5: Table S3). The GTGTTGGC, AAC, and CCATTC haplotypes of the blocks 1, 2 and 5 were significantly more frequent in controls than patients with *P* = 0.0075, 0.0414, and 0.0082, respectively, indicating its association with decreased disease risk. In contrast, the GCACTC and ACC haplotypes of the blocks 5 and 6 were significantly more frequent in the patients than the controls with *P* = 0.0103 and 0.0114, respectively, indicating the association with increased disease risk. After Bonferroni’s correction for multiple testing by multiplying *P*-value by the number of observed haplotypes in each block, the differences remained statistically significant only for the haplotypes GTGTTGGC, CCATTC and ACC of blocks 1, 5, and 6 (*P* = 0.03, 0.041, and 0.0456, respectively).

#### HAO1

All 35 SNPs on microarray located in *HAO1* gene region were analyzed for HWE and genotype and allele frequencies of the patient and control groups. Initially, 3 SNPs (rs1983560, rs2423331, and rs7271299) which were found to have low call rate of genotyping (less than 90%) in either the patient or control groups were disqualified. Two monomorphic SNPs (rs8124232 and rs16994134) were observed in both groups and were excluded from further statistical analysis. Allele frequencies of 11 SNPs showed significant differences (*P* < 0.05) between the patient and control groups (Table 5). Analysis of individual SNPs in the patient and control groups using the SNPStats program revealed significant differences of genotype frequencies of 10 SNPs. The model of inheritance considered by the least value of AIC was found to be dominant for the SNPs with significant differences. The patient group had significantly higher proportions of the alleles than that of the control group, indicating that associated alleles increase the risk of KSD (Table 5).

**Table 5:** **Genotype and allele frequencies of SNPs in *HAO1 *** **gene observed in 105 control subjects and 101 patients**

**SNP ID**	**Genotype**	**Genotype frequency (%)**		**OR (95% CI)**	*P*-value*	**Allele**	**Allele frequency (%)**		**OR (95% CI)**	*P*-value*
		**Control**	**Patient**				**Control**	**Patient**		
rs6055363	A/A	44.8	32	1.00	0.06	A	67.6	57	1.00	**0.0265**
	A/G, G/G	55.2	68	1.72 (0.97-3.04)		G	32.4	43	1.57 (1.05-2.36)	
rs2294305	A/A	43.8	31	1.00	0.058	A	66.7	56.5	1.00	**0.0343**
	A/G, G/G	56.2	69	1.74 (0.98-3.08)		G	33.3	43.5	1.54 (1.03-2.30)	
rs2235250	G/G	44.8	31.7	1.00	0.053	G	68.1	55.9	1.00	**0.0110**
	G/A, A/A	55.2	68.3	1.75 (0.99-3.09)		A	31.9	44.1	1.68 (1.12-2.51)	
rs6077285	C/C	28	46.9	1.00	**0.0057**	C	57.5	66.8	1.00	0.0555
	C/G, G/G	72	53.1	0.44 0(0.24-0.79)		G	42.5	33.2	0.67 (0.45-1.01)	
rs2294301	C/C	34.3	53.5	1.00	**0.0054**	C	60	69.8	1.00	**0.0373**
	C/G, G/G	65.7	46.5	0.45 0(0.26-0.80)		G	40	30.2	0.65 (0.43-0.98)	
rs2423326	G/G	34.3	54.5	1.00	**0.0035**	G	60	72.8	1.00	**0.0061**
	G/A, A/A	65.7	45.5	0.44 0(0.25-0.77)		A	40	27.2	0.56 (0.37-0.85)	
rs6118004	T/T	33.3	58	1.00	**0.0004**	T	60	74.5	1.00	**0.0018**
	T/C, C/C	66.7	42	0.36 0(0.21-0.64)		C	40	25.5	0.51 (0.34-0.78)	
rs2205818	A/A	35.2	57.4	1.00	**0.0013**	A	61.9	74.3	1.00	**0.0072**
	A/G, G/G	64.8	42.6	0.40 (0.23-0.71)		G	38.1	25.7	0.56 (0.37-0.86)	
rs2142697	T/T	34	56.6	1.00	**0.0012**	T	61.2	74.2	1.00	**0.0050**
	T/C, C/C	66	43.4	0.40 (0.22-0.70)		C	38.8	25.8	0.55 (0.36-0.83)	
rs6140463	G/G	35.2	58	1.00	**0.001**	G	61.4	74.5	1.00	**0.0046**
	G/A, A/A	64.8	42	0.39 (0.22-0.69)		A	38.6	25.5	0.54 (0.36-0.83)	
rs2235245	C/C	35.2	58.4	1.00	**0.0008**	C	61.4	75.2	1.00	**0.0026**
	C/G, G/G	64.8	41.6	0.39 (0.22-0.68)		G	38.6	24.8	0.52 (0.34-0.80)	
rs6086287	A/A	72.4	58.4	1.00	**0.035**	A	84.8	77.2	1.00	0.0510
	A/C, C/C	27.6	41.6	1.87 (1.04-3.34)		C	15.2	22.8	1.64 (0.99-2.70)	
rs2255183	T/T	71.4	56.4	1.00	**0.025**	T	84.3	76.2	1.00	**0.0399**
	T/C, C/C	28.6	43.6	1.93 (1.08-3.44)		C	15.7	23.8	1.67 (1.02-2.74)	

OR, odds ratio; CI, confidence interval.

*Uncorrected *P*-value.

Significant *P*-values are indicated in bold.

Three LD blocks spanning 17, 20, and 7 kb containing 5, 12, and 2 SNPs were defined from the genotyping data of 101 patients and 105 control subjects with 4, 5, and 2 observed haplotypes for blocks 1, 2, and 3, respectively (Additional file 6: Figure S3 and Additional file 7: Table S4). The GGGAC haplotype of the first block was significantly more frequent in the patients than in the controls with *P* = 0.0193 (OR 1.615, 95% CI 1.08- 2.414), indicating its association with increased disease risk. In contrast, the CAACGCTCAAAG and AT haplotypes of the second and the third blocks were significantly more frequent in the controls than in the patients with *P* = 0.0045 (OR 0.479, 95% CI 0.286-0.801) and 0.0399 (OR 0.598, 95% CI 0.365- 0.979), indicating the association with decreased disease risk. After Bonferroni’s correction for multiple testing by multiplying *P*-value by the number of observed haplotypes in each block, the differences remained statistically significant only for the haplotype of block 2 (*P* = 0.0225).

## Discussion

Over the last decade, a number of studies have been conducted to identify genetic factors contributing to KSD. Candidate gene approach is an important tool for this endeavour. In the present candidate gene association study, initially conducted in 101 NE Thai patients and 105 control subjects, we identified 82 different SNPs in 22 genes that showed suggestive associations with KSD from the initial analysis of 104 reported candidate genes. Four genes, including *BGLAP*, *AHSG*, *CD44*, and *HAO1*, were selected as candidate genes of KSD in NE Thai population for further analyses since they carried SNPs with highly statistical differences (*P* < 0.005) of allele frequencies between the patient and control groups and high proportion of significant SNPs within the gene.

*BGLAP* gene encodes osteocalcin or bone gamma-carboxyglutamate (gla) protein. This vitamin K-dependent protein is related to bone resorption and may change blood levels of calcium ions [25]. In mouse, osteocalcin has a similar pattern of expression and identical structural features to nephrocalcin, a calcium-binding protein partially purified from kidney that plays a role in calcium reabsorption and in prevention of nephrolithiasis [26]. Therefore, osteocalcin might associate with kidney stone disease. However, the *Hind* III polymorphism located at the promoter region was not correlated with calcium oxalate stone disease in Taiwanese patients [27]. In the present study, rs759330 located 144 bp downstream of *BGLAP* gene showed significant differences of allele frequency between the 216 patients and 216 control subjects with *P* = 0.0001. This result indicates the association of *BGLAP* and the disease; however, the functional SNP could not be identified. Only one exonic SNP, rs182775321, was detected in *BGLAP* by DNA sequencing but it showed no significant differences of genotype and allele frequencies between the patient and control groups. Interestingly, while we were looking for other candidate genes close to rs759330 that may be involved in the disease, we found that this SNP is located in the 3′UTR of *PAQR6* gene encoding progestin and adipoQ receptor family member VI. Prediction for miRNA binding site by using MicroCosm Targets at EMBL-EBI (http://www.ebi.ac.uk/enright-srv/microcosm/htdocs/targets/v5/) revealed that rs759330 is located at the binding site of hsa-miR-424*. The entire sequence of predicted binding site for hsa-miR-424* at 3′UTR of *PAQR6* gene including the position of rs759330 is shown (Additional file 8: Figure S4).

*PAQR6* has been reported to have progestin-binding characteristics [28]. The kidney was found to be the site of receptors for progesterone [29] and progesterone can stimulate Ca^2+^ reabsorption at distal part of the nephron [30]. The level of *PAQR6* expression may correlate with progesterone that stimulates Ca^2+^ reabsorption in the kidney and it may be involved in pathogenesis of KSD. In the present study, we found that the patient group had significantly higher proportions of C allele of rs759330 than the control group, indicating that C allele was susceptible to KSD. Since rs759330 is located at binding site of hsa-miR-424*, the presence of rs759330-C allele may decrease *PAQR6* expression leading to reduction of progesterone receptors in the kidney. The reduction of progesterone molecule which associates with decreased level of Ca^2+^ reabsorption may increase risk of KSD. For this reason, rs759330 may affect miRNA binding resulting in mRNA expression level. The relationship between *PAQR6* and KSD requires further investigation.

*AHSG* gene encodes fetuin-A or alpha-2-HS-glycoprotein, a circulating calcium-regulatory glycoprotein that inhibits extraosseous calcification. It has been reported that the patients with urolithiasis had lower urinary fetuin-A levels compared with that of healthy subjects [31]. In addition, two *AHSG* gene polymorphisms, rs4917 and rs4918, were evaluated in 103 Turkish patients with calcium oxalate nephrolithiasis and 73 healthy subjects. The results revealed that rs4918 was associated with higher risk for renal calcium oxalate stone formation [32]. In our study, 3 SNPs on microarray were initially analyzed and the allele and genotype frequencies of rs2070634 and rs2070635 were significantly different between the patient and control groups (Table 3). Two *AHSG* haplotypes were slightly associated with kidney stone risk, haplotype AGC with decreased disease risk and haplotype ATT with increased disease risk. However, the associations of both haplotypes were not statistically significant after correction. Two non- synonymous SNPs, rs4917 and rs4918, were also genotyped in 112 each of patient and control groups but no significant differences of allele and genotype frequencies between the patient and control groups were observed. This result is different from that of the previous study, showing that rs4918 was associated with KSD in Turkish patients [31]. Our result demonstrated that rs4918 was not associated with kidney stone formation in the population studied (*P* = 0.66, OR 0.78, 95% CI 0.51-1.18). The different result may be attributable to genetic diversity among these two populations and also the small numbers of patients and control subjects included in the present study.

*CD44* gene encodes CD44 molecule. This transmembrane protein is a receptor for hyaluronic acid (HA) and can also interact with osteopontin (OPN), a major component in the urinary stone matrix that inhibits nucleation, growth, and aggregation of CaOx crystals and also reduces binding of crystals to renal epithelial cells in *vitro*[33-36]. In a study in rat, the expression of HA, OPN, and CD44 by injured/regenerating tubular cells seems to play a role in crystal retention in the kidney [37]. Thus, CD44 may be a candidate gene of KSD. In this association study, genotype or allele frequencies of 13 SNPs in the *CD44* gene were significantly different between the patient and control groups (Table 4). Haplotypes associated with decreased and increased kidney stone risk were also identified but they were observed in a different haplotype block with different frequency (Additional file 5: Table S3). This study indicates the potency of *CD44* gene for KSD in the population studied. However, the variants causing the disease should be further investigated.

*HAO1* gene encodes glycolate oxidase 1. Human glycolate oxidase catalyzes flavin mononucleotide -dependent oxidation of glycolate to glyoxylate, and of glyoxylate to oxalate. The presence of glycolate oxidase 1 in liver and kidney peroxisomes and its ability to oxidize glyoxylate to oxalate, a key metabolite in the kidney stone formation, is of particular importance for individuals with primary hyperoxaluria type I, as a consequence of their inability to convert glyoxylate to glycine in the peroxisome [38-40]. In our study, we found 13 different SNPs, having either genotype or allele frequencies with significant differences between the patient and control groups (Table 5). Haplotypes associated with decreased and increased disease risks were also observed (Additional file 7: Table S4), indicating the potency of *HAO1* gene for KSD in the population studied although the variants associated with the disease should be further investigated.

Although a genome-wide association study (GWAS) was conducted in 101 patients and 105 control subjects in the beginning of this study, a potential variation could not be identified. Initially, 57 SNPs distributed on the genome were found to have statistical significance after correction by qualified 684,142 SNPs on microarray. Of these, 28 SNPs were located in the gene regions but the ratios of significant SNPs over non-significant SNPs within the same genes were low. In addition, only 6 SNPs were predicted to have functional impacts as examined by SNP Function Prediction (FuncPred, The National Institute of Environmental Health Sciences (NIEHS) http://snpinfo.niehs.nih.gov/snpinfo/snpfunc.htm) and when one SNP each in a gene of interest was selected to for validation, it was found to be false positive results. The unsuccessful analysis by GWAS in this study may be attributable to the small sample size leading to low statistical power (power 0.44 as calculated by Quanto, http://biostats.usc.edu/software). This led us to change the analysis method to be candidate gene association study. When an alternative approach by candidate gene association study of 104 reported candidate genes were taken, a number of potential variants were identified and a SNP rs759330 in *PAQR6* was validated in 216 each of patient and control groups by HRM method, the statistical power was increased to be 0.87. However, a larger sample size is required to validate the relationship of other candidate genes. Although a low-powered sample size is a limitation of this study, the association study based on the reported candidate genes has made it possible to identify the potential candidate genes and the associated SNPs for KSD in the studied population.

## Conclusions

In conclusion, our study suggests that *BGLAP* together with *PAQR6*, *AHSG*, *HAO1*, and *CD44* are potential candidates associated with KSD in NE Thai patients. Replication studies with a larger sample size are necessary to validate these associations. Of these, *PAQR6* comes into our view as the most potent candidate since the associated SNP rs759330 is located in the miRNA binding, which may affect mRNA expression level.

## Abbreviations

KSD: Kidney stone disease; SNP: Single nucleotide polymorphism; HRM: High-resolution melting; AIC: Akaike’s Information Criterion; OR: Odds ratio.

## Competing interests

The authors do not have any conflicts of interest, financial or otherwise, to declare.

## Authors’ contributions

NR and PY designed the study and wrote the article; NR, CN, NSu and OP carried out the molecular genetic studies and analyzed the data; NSa, SS, DC and PY collected the samples and participated in clinical data collection. All authors read and approved the final manuscript.

## Pre-publication history

The pre-publication history for this paper can be accessed here:

http://www.biomedcentral.com/1471-2350/15/50/prepub

## Supplementary Material

Additional file 1: Table S1 Candidate genes for kidney stone disease searched from PubMed, SNPs3D, and HuGE Navigator. Click here for file

Additional file 2: Figure S1 Linkage disequilibrium (LD) plots showing D’ and LD block of 3 genotyped SNPs in *AHSG* gene from 101 patients and 105 controls determined by the Haploview program. Click here for file

Additional file 3: Table S2 Association between haplotypes consisting of 3 SNPs of *AHSG* gene and kidney stone risk. Click here for file

Additional file 4: Figure S2 Linkage disequilibrium (LD) plots showing D’ and LD block of 49 genotyped SNPs in *CD44* gene from 101 patients and 105 controls determined by the Haploview program. Click here for file

Additional file 5: Table S3 Association between haplotypes consisting of 49 SNPs of *CD44* gene and kidney stone risk. Click here for file

Additional file 6: Figure S3 Linkage disequilibrium (LD) plots showing D’ and LD block of 30 genotyped SNPs in *HAO1* gene from 101 patients and 105 controls determined by the Haploview program. Click here for file

Additional file 7: Table S4 Association between haplotypes consisting of 30 SNPs of *HAO1* gene and kidney stone risk. Click here for file

Additional file 8: Figure S4 MicroRNA hsa-miR-424* binding site at 3′UTR of *PAQR6* gene and the position of rs759330. Click here for file
