# Supplementary material for: A whole genome SNP genotyping by DNA microarray and candidate gene association study for kidney stone disease
Source: BMC Med Genet. 2014 May 2;15:50. doi: 10.1186/1471-2350-15-50 (PMC4031563; doi:10.1186/1471-2350-15-50)
Supplement: Additional file 1: Table S1 — Candidate genes for kidney stone disease searched from PubMed, SNPs3D, and HuGE Navigator. [file 1471-2350-15-50-S1.docx]

**Additional file 1: Table S1. Candidate genes for kidney stone disease searched from** PubMed (P), SNPs3D (S), and HuGE Navigator (H)

| **No.** | **Gene** | **Chr.** | **Gene name** | **Reference** | | |
| --- | --- | --- | --- | --- | --- | --- |
|  |  |  |  | **P** | **S** | **H** |
| 1 | CLCNKB | 1 | chloride channel Kb | Y | Y |  |
| 2 | HSPG2 | 1 | heparan sulfate proteoglycan 2 |  |  | Y |
| 3 | CLDN19 | 1 | claudin 19 | Y |  |  |
| 4 | MAST2 | 1 | microtubule associated serine/threonine kinase 2 |  | Y |  |
| 5 | HAO2 | 1 | hydroxyacid oxidase 2 (long chain) |  | Y |  |
| 6 | S100A10 | 1 | S100 calcium binding protein A10 (calcitonin receptor) | Y |  |  |
| 7 | S100A8 | 1 | S100 calcium binding protein A8 (calgranulin A) | Y | Y | Y |
| 8 | S100A9 | 1 | S100 calcium binding protein A9 (calgranulin B) | Y |  | Y |
| 9 | S100A12 | 1 | S100 calcium binding protein A12 (calgranulin C) | Y |  | Y |
| 10 | BGLAP | 1 | bone gamma-carboxyglutamate (gla) protein (osteocalcin) | Y |  | Y |
| 11 | ADCY10 (SAC) | 1 | adenylate cyclase 10 (soluble) |  | Y |  |
| 12 | SLC26A9 | 1 | solute carrier family 26, member 9 |  | Y |  |
| 13 | SLC3A1 | 2 | solute carrier family 3, member 1 | Y | Y | Y |
| 14 | REG1B | 2 | regenerating islet-derived 1 beta |  | Y |  |
| 15 | REG1A | 2 | regenerating islet-derived 1 alpha; regenerating islet-derived 1 pseudogene |  | Y |  |
| 16 | REG1P | 2 | regenerating islet-derived 1 alpha; regenerating islet-derived 1 pseudogene |  | Y |  |
| 17 | REG3A | 2 | regenerating islet-derived 3 alpha |  | Y |  |
| 18 | GGCX | 2 | gamma-glutamyl carboxylase |  |  | Y |
| 19 | IL1R1 | 2 | interleukin 1 receptor, type I | Y |  | Y |
| 20 | IL1B | 2 | interleukin 1, beta | Y |  | Y |
| 21 | IL1RN | 2 | interleukin 1 receptor antagonist |  | Y | Y |
| 22 | FN1 | 2 | fibronectin 1 | Y |  | Y |
| 23 | AGXT | 2 | alanine-glyoxylate aminotransferase | Y | Y | Y |
| 24 | SLC26A6 | 3 | solute carrier family 26, member 6; cadherin |  | Y | Y |
| 25 | NIT2 | 3 | nitrilase family, member 2 |  | Y |  |
| 26 | CASR | 3 | calcium-sensing receptor | Y | Y | Y |
| 27 | TRPC1 | 3 | transient receptor potential cation channel | Y |  |  |
| 28 | AHSG | 3 | alpha-2-HS-glycoprotein (Fetuin-A) | Y |  | Y |
| 29 | CLDN16 | 3 | claudin 16 |  | Y |  |
| 30 | SLC26A1 | 4 | solute carrier family 26 (sulfate transporter), member 1 |  | Y |  |
| 31 | SLC2A9 | 4 | solute carrier family 2 (facilitated glucose transporter), member 9 |  |  | Y |
| 32 | SPP1 | 4 | secreted phosphoprotein 1 | Y | Y | Y |
| 33 | SLC12A2 | 5 | solute carrier family 12 (sodium/potassium/chloride transporters), member 2 |  | Y |  |
| 34 | SLC26A2 | 5 | solute carrier family 26 (sulfate transporter), member 2 |  | Y |  |
| 35 | PCDH24 (PCLKC) | 5 | protocadherin 24 |  | Y |  |
| 36 | SLC34A1 | 5 | solute carrier family 34 (sodium phosphate), member 1 | Y | Y |  |
| 37 | EDN1 | 6 | endothelin 1 |  | Y |  |
| 38 | TNF | 6 | tumor necrosis factor (TNF superfamily, member 2) |  |  | Y |
| 39 | TAP2 | 6 | transporter 2, ATP-binding cassette, sub-family B (MDR/TAP) | Y |  | Y |
| 40 | TAP1 | 6 | transporter 1, ATP-binding cassette, sub-family B (MDR/TAP) |  |  | Y |
| 41 | SLC26A8 | 6 | solute carrier family 26, member 8 |  | Y |  |
| 42 | VEGF (VEGFA) | 6 | vascular endothelial growth factor A | Y | Y | Y |
| 43 | ESR1 | 6 | estrogen receptor 1 |  |  | Y |
| 44 | SOD2 | 6 | superoxide dismutase 2, mitochondrial |  |  | Y |
| 45 | HNRNPA2B1 | 7 | heterogeneous nuclear ribonucleoprotein A2/B1 |  | Y |  |
| 46 | EGFR | 7 | epidermal growth factor receptor |  |  | Y |
| 47 | CALCR | 7 | calcitonin receptor |  |  | Y |
| 48 | TAC1 | 7 | tachykinin, precursor 1 |  | Y |  |
| 49 | SLC26A5 | 7 | solute carrier family 26, member 5 (prestin) |  | Y |  |
| 50 | SLC26A4 | 7 | solute carrier family 26, member 4 (pendrin) |  | Y |  |
| 51 | SLC26A3 | 7 | solute carrier family 26, member 3 |  | Y |  |
| 52 | CAV2 | 7 | caveolin 2 | Y |  |  |
| 53 | CAV1 | 7 | caveolin 1 | Y |  |  |
| 54 | TRPV6 | 7 | transient receptor potential cation channel, subfamily V, member 6 | Y |  | Y |
| 55 | TRPV5 | 7 | transient receptor potential cation channel, subfamily V, member 5 | Y | Y |  |
| 56 | NAT2 | 8 | N-acetyltransferase 2 (arylamine N-acetyltransferase) | Y |  | Y |
| 57 | ATP6V1B2 | 8 | ATPase, H+ transporting, lysosomal 56/58kDa, V1 subunit B2 |  | Y |  |
| 58 | SLC26A7 | 8 | solute carrier family 26, member 7 |  | Y |  |
| 59 | GRHPR | 9 | glyoxylate reductase/hydroxypyruvate reductase | Y | Y | Y |
| 60 | AMBP | 9 | alpha-1-microglobulin/bikunin precursor | Y | Y | Y |
| 61 | WAC | 10 | WW domain containing adaptor with coiled-coil |  | Y |  |
| 62 | ZNF365 | 10 | zinc finger protein 365 | Y | Y |  |
| 63 | PLAU | 10 | plasminogen activator, urokinase | Y |  | Y |
| 64 | HOGA1 | 10 | 4-hydroxy-2-oxoglutarate aldolase 1 | Y |  | Y |
| 65 | CYP17A1 | 10 | cytochrome P450, family 17, subfamily A, polypeptide 1 |  |  | Y |
| 66 | IGF2 | 11 | insulin-like growth factor 2 (somatomedin A) |  |  | Y |
| 67 | CD44 | 11 | CD44 molecule (Indian blood group) |  | Y |  |
| 68 | F2 | 11 | coagulation factor II (thrombin) | Y |  | Y |
| 69 | GSTP1 | 11 | glutathione S-transferase pi 1 | Y |  | Y |
| 70 | RASGRP2 | 11 | RAS guanyl releasing protein 2 (calcium and DAG-regulated) |  | Y |  |
| 71 | IL18 | 11 | interleukin 18 (interferon-gamma-inducing factor) | Y |  | Y |
| 72 | KCNJ1 | 11 | potassium inwardly-rectifying channel, subfamily J, member 1 | Y | Y |  |
| 73 | FGF23 | 12 | fibroblast growth factor 23 |  | Y |  |
| 74 | LPAR5 | 12 | lysophosphatidic acid receptor 5 |  | Y |  |
| 75 | MGP | 12 | matrix Gla protein | Y |  | Y |
| 76 | VDR | 12 | vitamin D (1,25- dihydroxyvitamin D3) receptor | Y | Y | Y |
| 77 | SDS | 12 | serine dehydratase |  | Y |  |
| 78 | ORAI1 | 12 | ORAI calcium release-activated calcium modulator 1 | Y |  | Y |
| 79 | KL | 13 | klotho | Y |  | Y |
| 80 | RASGRP1 | 15 | RAS guanyl releasing protein 1 (calcium and DAG-regulated) |  | Y |  |
| 81 | SLC12A1 | 15 | solute carrier family 12 (sodium/potassium/chloride transporters), member 1 | Y | Y |  |
| 82 | ANXA2 | 15 | annexin 2 | Y |  |  |
| 83 | CYP1A1 | 15 | cytochrome P450, family 1, subfamily A, polypeptide 1 | Y |  | Y |
| 84 | UMOD | 16 | uromodulin | Y | Y | Y |
| 85 | AMFR | 16 | autocrine motility factor receptor |  | Y |  |
| 86 | SLC12A3 | 16 | solute carrier family 12 (sodium/chloride transporters), member 3 |  | Y |  |
| 87 | CDH1 | 16 | cadherin 1, type 1, E-cadherin (epithelial) | Y | Y | Y |
| 88 | APRT | 16 | adenine phosphoribosyltransferase |  | Y |  |
| 89 | SLC13A2 | 17 | solute carrier family 13 (sodium-dependent dicarboxylate transporter), member 2 | Y |  | Y |
| 90 | RAPGEFL1 | 17 | Rap guanine nucleotide exchange factor (GEF)-like 1 |  | Y |  |
| 91 | WNK4 | 17 | WNK lysine deficient protein kinase 4 | Y | Y |  |
| 92 | SLC4A1 | 17 | solute carrier family 4, anion exchanger, member 1 |  | Y |  |
| 93 | SLC7A9 | 19 | solute carrier family 7 (cationic amino acid transporter, y+ system), member 9 | Y | Y | Y |
| 94 | TGFB1 | 19 | transforming growth factor, beta 1 |  | Y |  |
| 95 | HAO1 | 20 | hydroxyacid oxidase (glycolate oxidase) 1 |  | Y |  |
| 96 | CLDN14 | 21 | claudin 14 | Y |  | Y |
| 97 | TFF1 | 21 | trefoil factor 1 | Y |  | Y |
| 98 | PLP2 | X | proteolipid protein 2 (colonic epithelium-enriched) |  | Y |  |
| 99 | PRICKLE3 | X | prickle homolog 3 (Drosophila) |  | Y |  |
| 100 | CACNA1F | X | calcium channel, voltage-dependent, L type, alpha 1F subunit |  | Y |  |
| 101 | CLCN5 | X | chloride channel 5 | Y | Y | Y |
| 102 | SYP | X | synaptophysin |  | Y |  |
| 103 | AR | X | androgen receptor |  |  | Y |
| 104 | HPRT1 | X | hypoxanthine phosphoribosyltransferase 1 |  | Y |  |

Chr., chromosome: Y, yes.
