# Supplementary material for: A whole genome SNP genotyping by DNA microarray and candidate gene association study for kidney stone disease
Source: BMC Med Genet. 2014 May 2;15:50. doi: 10.1186/1471-2350-15-50 (PMC4031563; doi:10.1186/1471-2350-15-50)
Supplement: Additional file 3: Table S2 — Association between haplotypes consisting of 3 SNPs of AHSG gene and kidney stone risk. [file 1471-2350-15-50-S3.docx]

**Additional file 3: Table S2. Association between haplotypes consisting of 3 SNPs of *AHSG* gene and kidney stone risk.**

| **Haplotype** | **Frequency of haplotype** | | **OR**  **(95% CI)** | **χ2** | ***P*-value*** |
| --- | --- | --- | --- | --- | --- |
|  | **Control**  **(n = 105)** | **Patient**  **(n = 101)** |  |  |  |
| AGC | 0.652 | 0.545 | 0.637 (0.428 - 0.947) | 4.986 | **0.0256** |
| ATT | 0.186 | 0.285 | 1.749 (1.101 -2.779) | 5.693 | **0.0170** |
| TTT | 0.157 | 0.160 | 1.025 (0.604-1.656) | 0.007 | 0.9325 |

OR, odds ratio: CI, confidence interval.

* Uncorrected *P*-value

Significant *P* values are indicated in bold.
