# Supplementary material for: A whole genome SNP genotyping by DNA microarray and candidate gene association study for kidney stone disease
Source: BMC Med Genet. 2014 May 2;15:50. doi: 10.1186/1471-2350-15-50 (PMC4031563; doi:10.1186/1471-2350-15-50)
Supplement: Additional file 5: Table S3 — Association between haplotypes consisting of 49 SNPs of CD44 gene and kidney stone risk. [file 1471-2350-15-50-S5.docx]

**Additional file 5: Table S3. Association between haplotypes consisting of 49 SNPs of *CD44* gene and kidney stone risk.**

| **Haplotype** | **Frequency of haplotype** | | **OR**  **(95% CI)** | **χ2** | ***P*-value*** |
| --- | --- | --- | --- | --- | --- |
|  | **Control**  **(n = 105)** | **Patient**  **(n = 101)** |  |  |  |
| Block 1 |  |  |  |  |  |
| GTGTCAAT | 0.438 | 0.47 | 1.139 (0.772 - 1.679) | 0.431 | 0.5116 |
| ACGTCAAT | 0.29 | 0.337 | 1.24 (0.817 - 1.881) | 1.02 | 0.3125 |
| GTGTTGGC | 0.186 | 0.094 | 0.455 (0.253 - 0.818) | 7.151 | **0.0075** |
| GTACCGAC | 0.081 | 0.099 | 1.248 (0.634 - 2.456) | 0.411 | 0.5216 |
| Block 2 |  |  |  |  |  |
| GAC | 0.724 | 0.792 | 1.454 (0.922 - 2.291) | 2.611 | 0.1061 |
| AAC | 0.186 | 0.114 | 0.563 (0.323 - 0.983) | 4.158 | **0.0414** |
| AGA | 0.09 | 0.094 | 1.044 (0.535 - 2.034) | 0.016 | 0.9 |
| Block 3 |  |  |  |  |  |
| GA | 0.662 | 0.748 | 1.512 (0.987 - 2.318) | 3.621 | 0.057 |
| CA | 0.224 | 0.173 | 0.727 (0.446 - 1.184) | 1.65 | 0.199 |
| CT | 0.114 | 0.079 | 0.667 (0.343 - 1.296) | 1.445 | 0.2293 |
| Block 4 |  |  |  |  |  |
| CA | 0.871 | 0.916 | 1.606 (0.846 - 3.046) | 2.129 | 0.1445 |
| TG | 0.124 | 0.084 | 0.65 (0.341 - 1.239) | 1.732 | 0.1882 |
| Block 5 |  |  |  |  |  |
| GCACTC | 0.652 | 0.767 | 1.757 (1.14 - 2.709) | 6.59 | **0.0103** |
| CTGTAC | 0.124 | 0.069 | 0.527 (0.267 - 1.041) | 3.489 | 0.0618 |
| CCATTT | 0.09 | 0.079 | 0.865 (0.432 - 1.733) | 0.168 | 0.6817 |
| GCATTC | 0.062 | 0.069 | 1.128 (0.517 - 2.464) | 0.092 | 0.7615 |
| CCATTC | 0.067 | 0.015 | 0.211 (0.06 - 0.746) | 6.988 | **0.0082** |
| Block 6 |  |  |  |  |  |
| ACC | 0.324 | 0.445 | 1.675 (1.122 - 2.5) | 6.407 | **0.0114** |
| ACT | 0.323 | 0.286 | 0.841 (0.552 - 1.28) | 0.653 | 0.4191 |
| GTC | 0.319 | 0.252 | 0.721 (0.469 - 1.108) | 2.231 | 0.1353 |
| ATC | 0.028 | 0.005 | 0.186 (0.024 - 1.435) | 3.289 | 0.0697 |
| Block 7 |  |  |  |  |  |
| CACA | 0.857 | 0.856 | 0.994 (0.573 - 1.724) | 0.001 | 0.9811 |
| CGTG | 0.067 | 0.079 | 1.204 (0.572 - 2.536) | 0.237 | 0.6266 |
| AGTG | 0.071 | 0.044 | 0.606 (0.259 - 1.418) | 1.353 | 0.2448 |

OR, odds ratio; CI, confidence interval.

* Uncorrected *P*-value

Significant *P* values are indicated in bold.
