# Supplementary material for: A whole genome SNP genotyping by DNA microarray and candidate gene association study for kidney stone disease
Source: BMC Med Genet. 2014 May 2;15:50. doi: 10.1186/1471-2350-15-50 (PMC4031563; doi:10.1186/1471-2350-15-50)

**Additional file 6: Figure S3. Linkage disequilibrium (LD) plots showing D’ and LD block of 30 genotyped SNPs in *HAO1* gene from 101 patients and 105 controls determined by the Haploview program.** Genomic structure of *HAO1* gene and location of SNPs are indicated above the LD plot. Exons are indicated by black boxes and untranslated regions are represented in white. LD blocks are indicated by the black pentagon line. Squares represent LD and LOD score of between SNPs. Numbers in boxes represent D’ (x 100). Bottom left panel displays the frequency of haplotype and levels of recombination between the blocks. The strength of LD is indicated with the bottom right-color scheme.


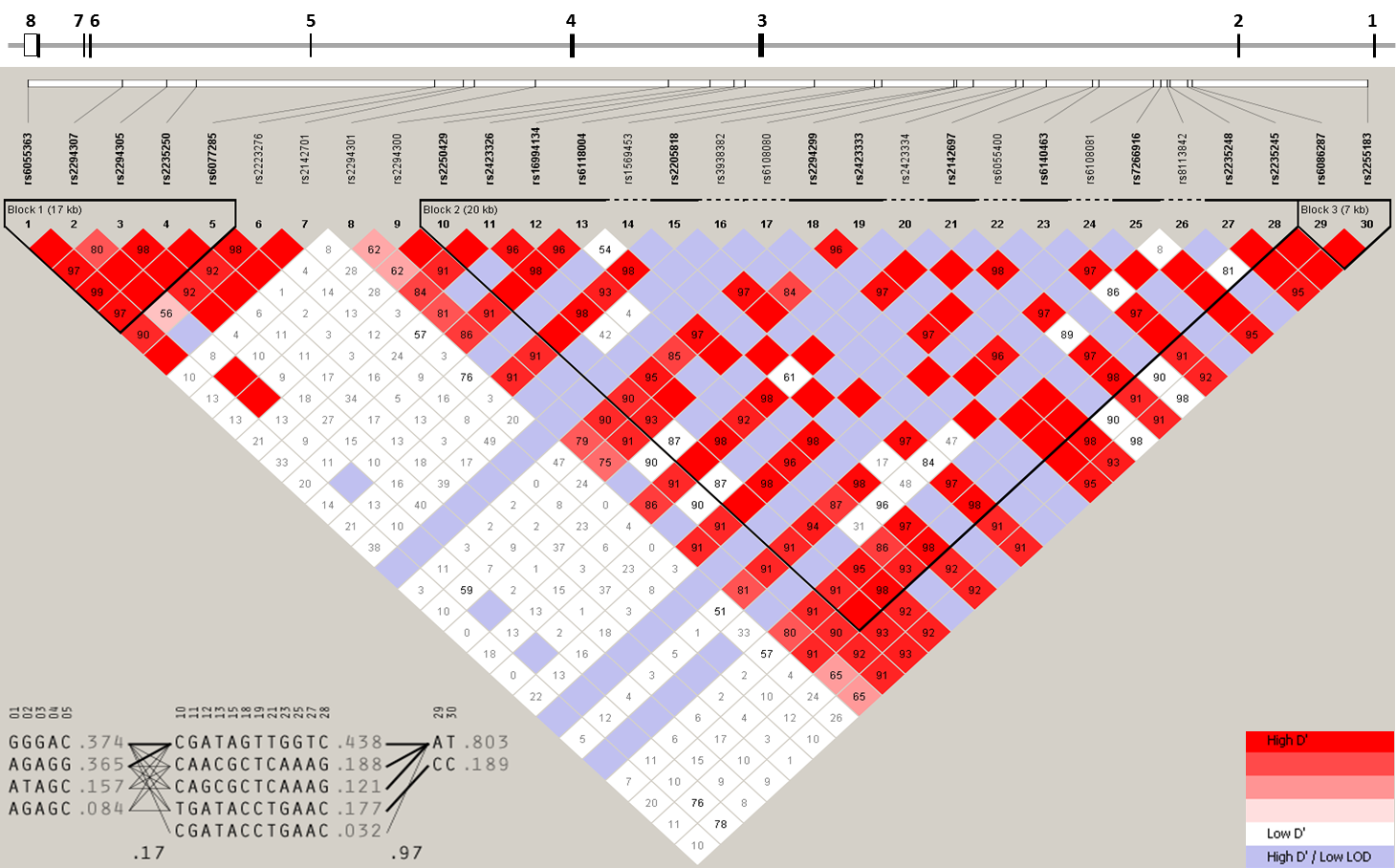

Supplement: Additional file 6: Figure S3 — Linkage disequilibrium (LD) plots showing D’ and LD block of 30 genotyped SNPs in HAO1 gene from 101 patients and 105 controls determined by the Haploview program. [file 1471-2350-15-50-S6.docx]
