# Supplementary material for: A whole genome SNP genotyping by DNA microarray and candidate gene association study for kidney stone disease
Source: BMC Med Genet. 2014 May 2;15:50. doi: 10.1186/1471-2350-15-50 (PMC4031563; doi:10.1186/1471-2350-15-50)
Supplement: Additional file 7: Table S4 — Association between haplotypes consisting of 30 SNPs of HAO1 gene and kidney stone risk. [file 1471-2350-15-50-S7.docx]

**Additional file 7: Table S4. Association between haplotypes consisting of 30 SNPs of *HAO1* gene and kidney stone risk.**

| **Haplotype** | **Frequency of haplotype** | | **OR**  **(95% CI)** | **χ2** | ***P*-value*** |
| --- | --- | --- | --- | --- | --- |
|  | **Control**  **(n = 105)** | **Patient**  **(n = 101)** |  |  |  |
| Block 1 |  |  |  |  |  |
| GGGAC | 0.319 | 0.431 | 1.615 (1.08- 2.414) | 5.472 | **0.0193** |
| AGAGG | 0.407 | 0.321 | 0.688 (0.459-1.03) | 3.337 | 0.0677 |
| ATAGC | 0.171 | 0.143 | 0.816 (0.479-1.39) | 0.576 | 0.4479 |
| AGAGC | 0.083 | 0.085 | 1.024 (0.511-2.052) | 0.006 | 0.938 |
| Block 2 |  |  |  |  |  |
| CGATAGTTGGTC | 0.401 | 0.476 | 1.356 (0.918-2.003) | 2.354 | 0.125 |
| CAACGCTCAAAG | 0.242 | 0.132 | 0.479 (0.286-0.801) | 8.058 | **0.0045** |
| TGATACCTGAAC | 0.147 | 0.207 | 1.511 (0.907-2.519) | 2.553 | 0.1101 |
| CAGCGCTCAAAG | 0.133 | 0.109 | 0.794 (0.438-1.441) | 0.576 | 0.4479 |
| CGATACCTGAAC | 0.038 | 0.025 | 0.654 (0.212-2.02) | 0.575 | 0.4484 |
| Block 3 |  |  |  |  |  |
| AT | 0.843 | 0.762 | 0.598 (0.365- 0.979) | 4.222 | **0.0399** |
| CC | 0.152 | 0.228 | 1.64 (0.995- 2.704) | 3.808 | 0.051 |

OR, odds ratio; CI, confidence interval.

* Uncorrected *P*-value

Significant *P* values are indicated in bold.
