# Supplementary material for: A whole genome SNP genotyping by DNA microarray and candidate gene association study for kidney stone disease
Source: BMC Med Genet. 2014 May 2;15:50. doi: 10.1186/1471-2350-15-50 (PMC4031563; doi:10.1186/1471-2350-15-50)

**Additional file 8: Figure S4. MicroRNA hsa-miR-424* binding site at 3’UTR of *PAQR6* gene and the position of rs759330.** Prediction for miRNA binding site by using MicroCosm Targets revealed that rs759330 is located at the binding site of hsa-miR-424*. Top and bottom sequences are 3’UTR of *PAQR6* where the SNP rs759330 alleles C and U (T) are represented in blue and red. Middle sequence is hsa-miR-424*. It was predicted to bind more strongly with rs759330-C than rs759330-U. Complementary base-pairing is denoted by the bars.


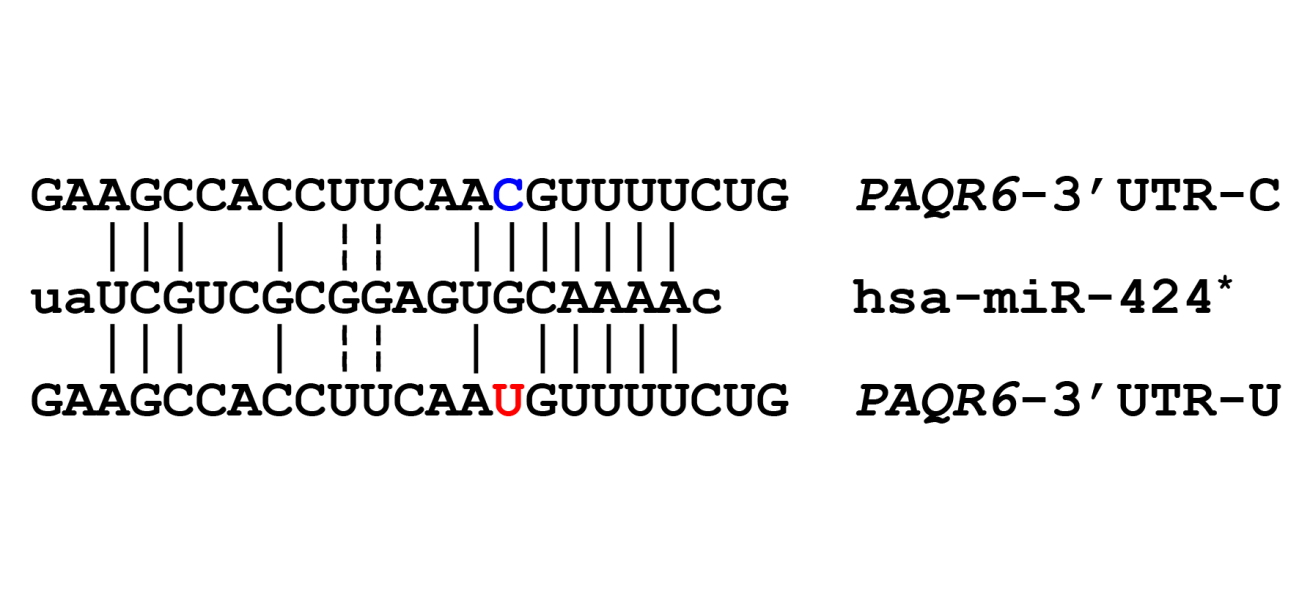

Supplement: Additional file 8: Figure S4 — MicroRNA hsa-miR-424* binding site at 3′UTR of PAQR6 gene and the position of rs759330. [file 1471-2350-15-50-S8.docx]
